# Effect of Interlayer Temperature-Controlled Thermal Cycling on the Microstructure and Mechanical Properties of Wire Arc Directed Energy Deposition H13 Steel

**DOI:** 10.3390/ma19010111

**Published:** 2025-12-29

**Authors:** Chuang Li, Hawke Suen, Yajin Yang, Liang Zhang, Qiuxia Chen, Tianlong Gao, Bo Yuan, Lyusha Cheng, Zhe Lv

**Affiliations:** 1 School of Materials and Metallurgy, University of Science and Technology Liaoning, Anshan 114051, China; lc042907@163.com; 2 Institute of Intelligent Manufacturing Technology, Shenzhen Polytechnic University, Shenzhen 518055, China; zhangliang@szpu.edu.cn (L.Z.); gtlzs18@163.com (T.G.); yuanbo@szpu.edu.cn (B.Y.); 3 Shenzhen Institute of Advanced Technology, Chinese Academy of Sciences, Shenzhen 518055, China; 4 School of Science, Harbin Institute of Technology, Shenzhen 518055, China; yangyajin1120@126.com; 5 School of Artificial Intelligence, Shenzhen Polytechnic University, Shenzhen 518055, China; chenqiuxia@szpu.edu.cn; 6 Department of Mechanical and Electrical Engineering, Shenzhen Polytechnic University, Shenzhen 518055, China

**Keywords:** WA-DED, H13 steel, interlayer temperature, finite element simulation, mechanical properties

## Abstract

Wire arc directed energy deposition (WA-DED) is a cost-effective technique for fabricating large metallic components. However, the inherent layer-by-layer deposition process leads to substantial heat accumulation, which significantly influences the resulting microstructure and mechanical properties. In this study, the effects of thermal cycling histories, at different interlayer temperatures, on the microstructural evolution and mechanical behavior of WA-DED fabricated H13 steel thin walls were systematically investigated, using an experimentally calibrated transient thermal model combined with experimental validation. Microstructural analysis revealed that at an interlayer temperature of 200 °C, the deposited material primarily consisted of coarse martensite with a low dislocation density and relatively large precipitates at a moderate volume fraction, resulting in an ultimate tensile strength of 1103 ± 28 MPa and an elongation of 14.6%. Increasing the interlayer temperature to 400 °C facilitated the formation of finer martensite with a higher dislocation density and smaller precipitates of slightly increased volume fraction. These microstructural refinements enhanced the tensile strength to 1549 ± 43 MPa, albeit at the expense of ductility, reducing elongation to 8.3%. When the interlayer temperature was further raised to 600 °C, fine martensite and a moderate dislocation density were retained; however, precipitate coarsening and a reduced volume fraction led to a decline in tensile strength to 1434 ± 33 MPa, accompanied by a slight recovery in elongation to 8.6%. Quantitative analysis based on classical strengthening models confirmed that dislocation strengthening is the dominant mechanism governing the variation in mechanical properties with changing interlayer temperature.

## 1. Introduction

H13 hot-work die steel is a critical material for hot-working tools in the mold industry owing to its excellent hardenability and superior comprehensive mechanical properties at elevated temperatures [1,2,3]. Conventional mold manufacturing primarily relies on casting followed by machining, yet it remains constrained by the limited capability of traditional forging techniques to produce geometrically complex components [4,5]. Furthermore, these conventional manufacturing routes for H13 steel are often characterized by low material utilization efficiency and prolonged production cycles, which collectively hinder its large-scale adoption and restrict further technological advancement. Consequently, the development of next-generation manufacturing processes is essential to overcome these limitations and broaden the application scope of H13 steel [6].

Additive manufacturing (AM), an emerging manufacturing technology, fabricates three-dimensional components by successively depositing metallic wire or powder feedstock in a layer-by-layer manner [7,8]. Its primary advantages lie in the significantly enhanced design freedom and material utilization efficiency, making it particularly suitable for producing geometrically complex and customized parts [9]. Compared to the conventional subtractive and formative manufacturing processes, AM offers remarkable benefits in terms of performance, costs, and productivity [10,11]. In recent years, extensive research has been devoted to various AM technologies, such as laser powder bed fusion (LPBF), electron beam directed energy deposition (EB-DED), and wire arc directed energy deposition (WA-DED) [10,12]. Among these, WA-DED employs metallic wire feedstock and an electric arc as the energy source [13,14]. This process can be implemented using different arc-based methods, including gas metal arc welding (GMAW) [15], gas tungsten arc welding (GTAW) [16], and plasma arc welding (PAW) [17]. Compared to LPBF and EB-DED processes, the WA-DED exhibits distinct advantages in deposition rate, cost-effectiveness, environmental adaptability, and material utilization [18].

The high deposition rate and cost-effectiveness of WA-DED have attracted significant research interest for the fabrication of metallic components. Dinovitzer et al. [19] identified welding current and travel speed as critical parameters governing the weld geometry and microstructure of nickel alloy deposits. However, the heat accumulation during continuous deposition significantly reduces the cooling rate, which can lead to the formation of undesirable microstructures. The inherent thermal cycling in multi-layer deposition causes progressive heat accumulation, thereby altering the thermal history and promoting non-equilibrium phase transformations and the formation of brittle phases. Consequently, both the dimensional accuracy and mechanical properties of the fabricated components are adversely affected. Therefore, precise control of the interpass temperature is widely recognized as a critical process parameter for effectively managing the thermal cycle, optimizing the microstructure, and enhancing the final properties. Rodrigues et al. [20] investigated the influence of heat input on the microstructure and mechanical properties of WA-DED fabricated high-strength low-alloy steel, demonstrating that the lower heat input suppresses excessive grain growth and produces finer grains. Ali et al. [21] showed that maintaining the interpass temperature above the martensite start temperature (Ms) enables the production of WA-DED fabricated hot-work tool steel with uniform hardness distribution along the build height. Li et al. [22] examined the effects of different thermal cycling histories on the microstructure and properties of the same material, revealing that faster interpass cooling promotes a more uniform distribution of precipitates. Xiong et al. [23] systematically investigated the effect of interpass temperature on the microstructure and mechanical properties of WA-DED-manufactured M300 steel, confirming that precise control of interpass temperature is not only essential for optimizing surface formation quality but also a key factor governing microstructural evolution. Therefore, the process parameters, particularly the interpass temperature, serve as effective means of minimizing heat accumulation, refining the microstructure, and improving the mechanical performance of WA-DED components.

This study aimed to investigate the effect of interlayer temperature on the microstructure of H13 steel fabricated via WA-DED. A robot-assisted gas-shielded metal arc welding system was employed to fabricate thin-walled H13 components. The effects of interlayer temperatures (200 °C, 400 °C, and 600 °C) on microstructural evolution and mechanical properties were systematically examined, supported by finite element simulations of the corresponding thermal histories. The results elucidate the intrinsic relationship between interlayer temperature, microstructural development, and macroscopic mechanical performance. This work provides essential process insights and a theoretical foundation for the fabrication of high-performance H13 tool steel components via WA-DED.

## 2. Experimental Procedures

### 2.1. Materials and Manufacturing Processes

Commercially available AISI H13 solid welding wire of a diameter of 1.2 mm was used in this study. Annealed H13 steel plates served as the substrate, with dimensions of 200 mm × 150 mm × 8 mm (length × width × thickness). The chemical composition of the substrate is detailed in Table 1. Prior to deposition, the substrate surfaces were first mechanically polished using a grinding machine and subsequently degreased with acetone to eliminate oxides and other contaminants. The H13 specimens were deposited using the process parameters summarized in Table 2. The key aspect of this experiment is maintaining the interlayer temperature at a prescribed value by regulating the dwell time after each deposited layer; deposition of the subsequent layer begins immediately once the temperature reaches the set point.

**Figure 1:**
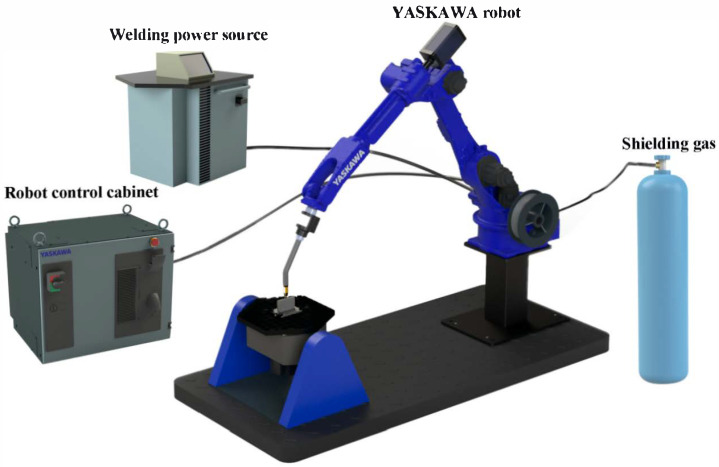
Schematic diagram of the WA-DED experimental system.

The GMAW technology was employed to fabricate H13 specimens in this study. The system primarily comprised a welding machine (SKS FP8i, Winsen, Germany) operating in MicroMIG-CC mode as the power source, a six-axis robot (MOTOMAN AR-2010, Yaskawa, Kitakyushu, Japan) for path planning, and an infrared thermometer for monitoring interlayer temperature. The experimental setup for the WA-DED process is illustrated in Figure 1.

### 2.2. Microstructural Characterization Methods

**Figure 2:**
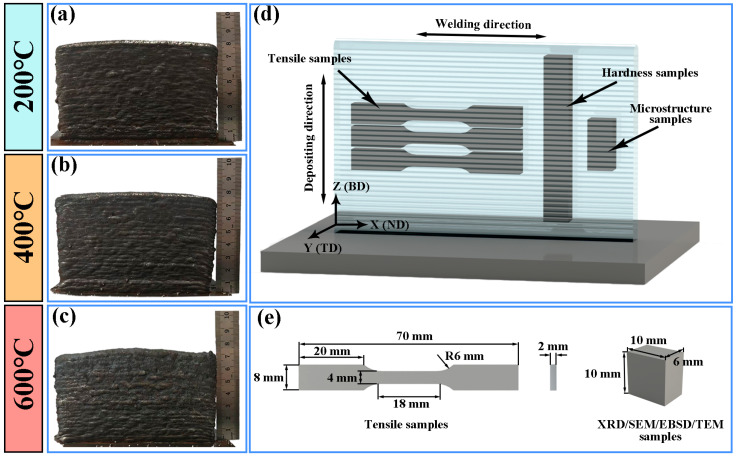
Macroscopic morphology and dimensional profiles of the samples fabricated at interlayer temperatures of 200 °C, 400 °C, and 600 °C: (**a**–**c**) sample macrographs; (**d**) sample cutting location; (**e**) specimen dimensional specifications.

The physical appearances of the deposited samples at interlayer temperatures of 200 °C, 400 °C, and 600 °C are shown in Figure 2a–c, respectively. As illustrated in Figure 2d,e, microscopic specimens for detailed examination were extracted from the thin-walled components using the wire electrical discharge machining (WEDM). Subsequently, the samples were characterized using X-ray diffraction (XRD, Bruker D8 Advance, Billerica, MA, USA), field-emission scanning electron microscopy (SEM, Gemini SEM 300, Oberkochen, Germany), SEM equipped with an electron backscatter diffraction (EBSD) detector (Oxford Symmetry, Abingdon, UK), and field-emission transmission electron microscopy (TEM, FEI Tecnai G2 F30, Hillsboro, OR, USA). The XRD analysis of the surface phase composition was performed using a Cu-Kα radiation source (λ = 1.5406 Å) over a scanning angle range of 20° to 110°, with a step size of 0.02° and a step time of 0.2 s. The etched samples were subsequently cleaned and dried with ethanol. Prior to EBSD analysis, the samples were electrochemically polished in an electrolyte solution (90 mL 95% C_2_H_5_OH + 10 mL 30% HClO_4_) at 30 V for 20 s. TEM specimens were mechanically thinned to approximately 50 μm, followed by electrochemical polishing at 253 K in an electrolyte solution (33 vol% HNO_3_ + 67 vol% CH_3_OH) under a working current of 12 A.

### 2.3. Mechanical Property Tests

The specific dimensions of the hardness and tensile test specimens are presented in Figure 2d,e. All the specimens were prepared using WEDM. Microhardness measurements were performed using a microhardness tester (HXS-1000TAC, Shanghai, China). Test points were selected at 2.5 mm intervals along the specimen height, from bottom to top. The Vickers indentation method was employed with a load of 0.1 kg and a dwell time of 15 s to determine the microhardness at each point. To minimize random errors and improve data reliability, each measurement point was tested three times, and the mean value was reported as the experimental result. Tensile tests were carried out at room temperature using a universal testing machine (SANS CMT5305, Eden Prairie, MN, USA) equipped with a laser extensometer, with a loading rate of 1.00 mm/min. For each condition, the average value of three independent specimens was reported as the final result. Additionally, the fracture surfaces of the tensile specimens were examined using SEM to analyze the fracture morphology and determine the material fracture mode.

## 3. Finite Element Simulation

Given the complex thermal history inherent to the WA-DED process, the heat accumulation affects previously deposited layers, consequently influencing the microstructure and mechanical properties of the component. Therefore, in this study, Abaqus 2022 finite element software was employed to establish a simulation model capable of replicating the thermal history of the entire manufacturing process.

According to Fourier’s law of heat conduction [24], transient heat transfer in the finite element method is governed by the three-dimensional heat conduction equation, which can be expressed as follows:

(1) $$cp\frac{\partial T_{t}}{\partial t}=\frac{\partial }{\partial x}(\lambda \frac{\partial T_{t}}{\partial x})+\frac{\partial }{\partial y}(\lambda \frac{\partial T_{t}}{\partial y})+\frac{\partial }{\partial z}(\lambda \frac{\partial T_{t}}{\partial z})+\overset{-}{Q},$$

where *c* denotes the specific heat, *ρ* is the density, *λ* is the thermal conductivity, *T_t_* is the temperature, *Q* is the heat input, and *t* is the time.

Employing the “element birth and death technique”, the thermal behavior during additive manufacturing was dynamically simulated by sequentially activating elements along the path of the moving heat source. In the WA-DED process, the primary modes of heat exchange between the component and its surroundings are convection and radiation. This combined heat transfer effect is characterized by the heat transfer coefficient, *α* [25]:

(2) $$\alpha =\frac{\varepsilon_{em}\sigma_{bol}\left(T_{tv}^{4}-T_{amb}^{4}\right)}{T_{tv}-T_{amb}}+\alpha_{con},$$

where *ε_em_* denotes the emissivity, *σ_bol_* is the Stefan–Boltzmann constant, *T_tv_* is the temperature variable, *T_amb_* is the ambient temperature, and *α_con_* is the convective heat transfer coefficient.

During the WA-DED deposition process, the dynamic movement of the heat source leads to an asymmetric power distribution along its path. Typically, the front half-ellipsoidal region exhibits a higher power density, whereas the rear half displays a lower power output, with each half corresponding to a quarter-ellipsoid. Therefore, a double-ellipsoidal heat source model is employed, as described by the following equations [26]:

(3) $$Q_{\mathrm{front}}\left(x,y,z\right)=\frac{6\sqrt{3}fQ_{p}}{a_{\mathrm{front}}bc\pi^{3/2}}\left[-\left(3\frac{x^{2}}{a_{\mathrm{front}}^{2}}+3\frac{y^{2}}{b^{2}}+3\frac{z^{2}}{c^{2}}\right)\right],$$

(4) $$Q_{\mathrm{rear}}\left(x,y,z\right)=\frac{6\sqrt{3}\left(2-f\right)Q_{p}}{a_{\mathrm{rear}}bc\pi^{3/2}}\left[-\left(3\frac{x^{2}}{a_{\mathrm{rear}}^{2}}+3\frac{y^{2}}{b^{2}}+3\frac{z^{2}}{c^{2}}\right)\right],$$

where *Q_p_* represents the arc heat source power, and *f* denotes the energy distribution coefficient for the two ellipsoidal sections, with a value of 0.86. *a_front_* is the semi-major axis of the front ellipsoid (3.8 mm), *a_rear_* is the semi-major axis of the rear ellipsoid (7.6 mm), *b* is the semi-minor axis (3.85 mm), and *c* is the molten pool depth (1.85 mm).

**Figure 3:**
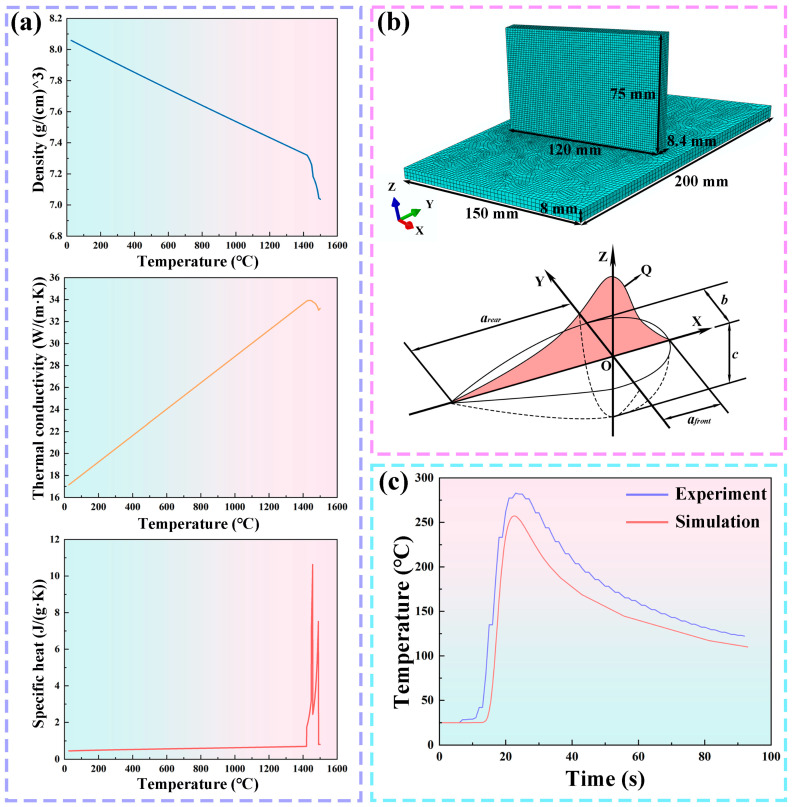
Models and parameters utilized in the simulation: (**a**) thermal–physical properties of materials; (**b**) mesh model and double-ellipsoidal heat source; (**c**) validation results of the finite element model.

As illustrated in Figure 3a, the thermophysical parameters of the simulated material were obtained using JMatPro software (2012). The material properties, including density, thermal conductivity, and specific heat capacity, were treated as temperature-dependent. Figure 3b shows the computational mesh model employed in this study, together with a schematic of the double-ellipsoidal heat source. To enhance computational efficiency, a transition mesh was adopted in the weld region. The final finite element model consisted of 45,723 DC3D8 (8-node hexahedral) elements and 57,786 nodes. To validate the model, a single deposition experiment was conducted. Thermal monitoring was performed using a multi-channel temperature recorder (MT500P, Shenzhen, China) coupled with a K-type thermocouple with an accuracy of ±0.1 °C. The thermocouple was positioned at 8 mm beneath the substrate to record the thermal history in real time during deposition. A comparison between the experimentally measured temperature curve at this location and the simulated result is presented in Figure 3c. The simulation results exhibit close agreement with the experimental measurements, thereby confirming the reliability of the model.

**Figure 4:**
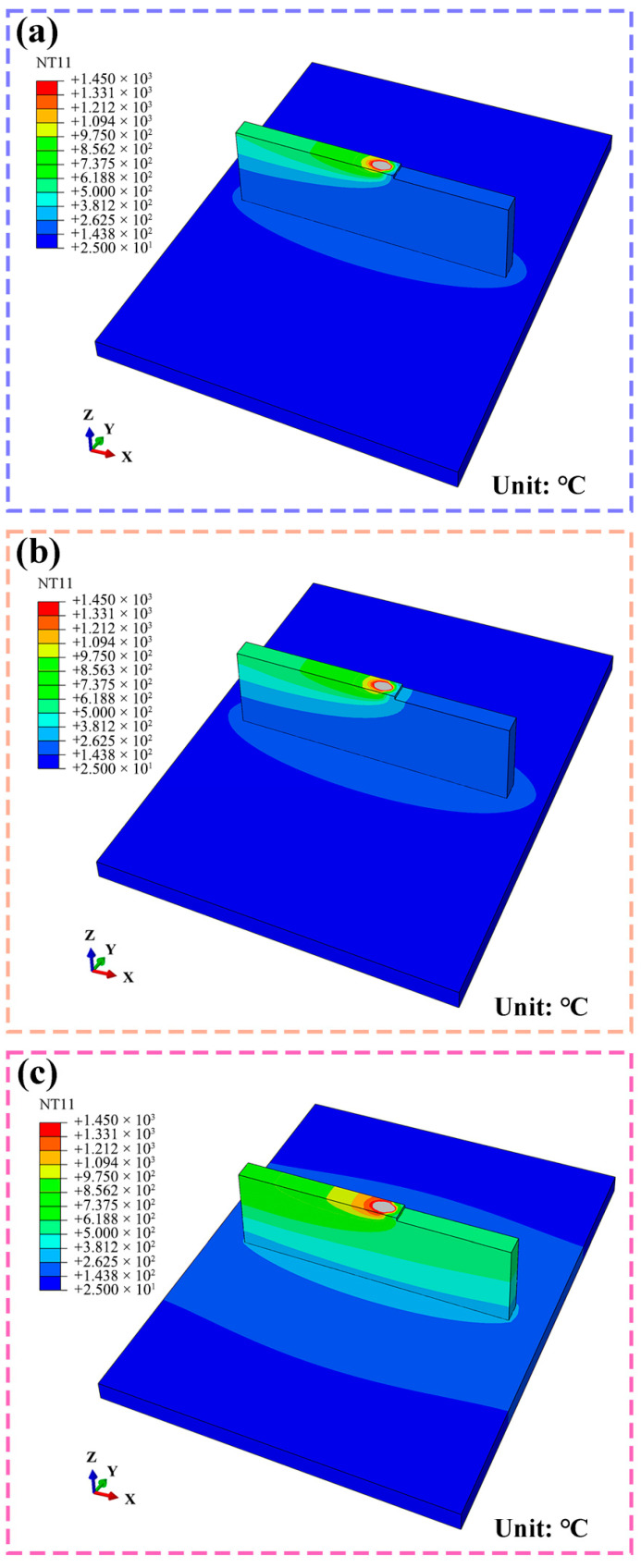
Simulated transient thermal field in the 25th layer at interlayer temperatures of (**a**) 200 °C, (**b**) 400 °C, and (**c**) 600 °C.

Figure 4 illustrates the three-dimensional spatial distribution of the transient temperature field within the component when the heat source reaches the midpoint of the 25th layer. The gray region corresponds to the molten pool, where the temperature exceeds the melting point of H13 steel (1450 °C). The molten pool and the heat-affected zone (HAZ) evolve synchronously with the moving heat source, exhibiting a spatially symmetrical and uniform double-ellipsoidal shape, characterized by a compressed leading edge and an elongated trailing edge. The interlayer temperature significantly affects the degree of heat accumulation. Compared to an interlayer temperature of 200 °C, conditions at 400 °C and 600 °C result in a wider HAZ and a lower temperature gradient along the deposition direction. This reduction in thermal gradient is attributed to the accumulation of heat from successive deposition passes, which decreases the overall cooling rate. Consequently, these distinct thermal distributions under different interlayer temperatures govern the subsequent solidification behavior and ultimately determine the evolution of the final microstructure.

## 4. Results

### 4.1. Effect of Interlayer Temperature on Microstructure

**Figure 5:**
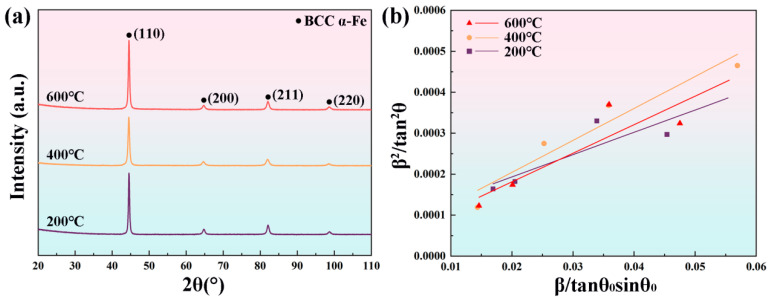
(**a**) XRD patterns of H13 steel specimens processed at different interlayer temperatures; (**b**) integral width analysis for determining average grain size and lattice microstrain in coherent diffraction regions.

Figure 5a presents the XRD patterns of samples processed at different interlayer temperatures. Only diffraction peaks corresponding to martensite (α) are observed, and the absence of peaks associated with retained austenite (γ_R_) indicates its low content. Similarly, carbides within the samples are undetectable by XRD due to their low volume fraction and small size. The dislocation density can be further quantified based on the XRD results. Dislocations in the material are classified into two types according to their storage mechanisms: statistically stored dislocations (SSDs) and geometrically necessary dislocations (GNDs) [27]. Using the integral breadth analysis method based on Bragg diffraction, the average grain size and lattice microstrain within coherently diffracting domains can be determined. As described in reference [28], the broadening of diffraction peaks, resulting from the grain size effects and lattice microstrain, can be approximated by a Cauchy function and a Gaussian function, respectively, leading to the following equation:

(5) $$\frac{\beta^{2}}{{tan}^{2}\theta_{0}}=\frac{\lambda }{D}\left(\frac{\beta }{tan\theta_{0}sin\theta_{0}}\right)+25\varepsilon^{2},$$

where *β* is the integral breadth of the four diffraction peaks (110), (200), (211), and (220) corresponding to the martensite phase in the XRD spectrum, *θ*_0_ is the peak position of the selected diffraction peak, *λ* is the radiation wavelength, *D* is the average grain size, and *ε* represents the lattice microstrain. The values of *D* and *ε* can be determined from the slope and intercept of the fitted line shown in Figure 5b. Subsequently, the total dislocation density, *ρ*, is calculated using the following equation [29]:

(6) $$\rho =\frac{2\sqrt{3}{\left(\varepsilon^{2}\right)}^{1/2}}{D\times b},$$

where *b* is the Burgers vector (0.248 nm [30]). The calculated dislocation density *ρ* results are as follows: 6.23 × 10^14^ m^−2^ at an interlayer temperature of 200 °C, 9.07 × 10^14^ m^−2^ at an interlayer temperature of 400 °C, and 8.21 × 10^14^ m^−2^ at an interlayer temperature of 600 °C.

**Figure 6:**
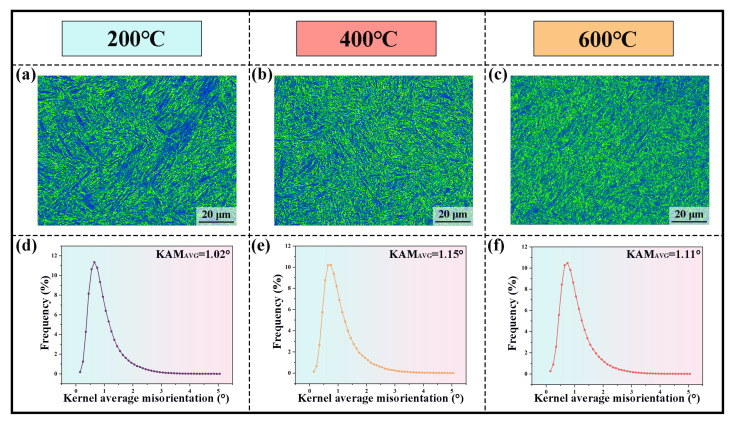
KAM images of H13 steel samples processed at different interlayer temperatures: (**a**,**d**) 200 °C; (**b**,**e**) 400 °C; (**c**,**f**) 600 °C.

To further characterize the dislocation density of samples prepared at different interlayer temperatures, EBSD analysis was conducted (Figure 6). Figure 6a–c present the distribution maps of the average kernel average misorientation (KAM) obtained from the EBSD. Since the KAM value is positively correlated with the local lattice distortion, these maps provide an intuitive representation of the spatial distribution of dislocations. The analysis indicates that the sample processed at an interlayer temperature of 200 °C exhibits a relatively low dislocation density across the observed region. In contrast, samples fabricated at interlayer temperatures of 400 °C and 600 °C show significantly higher dislocation densities. This trend is quantitatively corroborated by the average KAM values shown in Figure 6d–f, where the sample at 200 °C exhibits the lowest average KAM value of 1.02°, whereas samples processed at 400 °C and 600 °C display higher values of 1.15° and 1.11°, respectively. Notably, the sample treated at 400 °C shows the most pronounced increase. These results demonstrate that variations in interlayer temperature induce different thermal cycling histories during fabrication, leading to substantial differences in dislocation density. High-density dislocations act as effective barriers to dislocation motion and the initiation of plastic deformation, thereby significantly impeding dislocation mobility.

**Figure 7:**
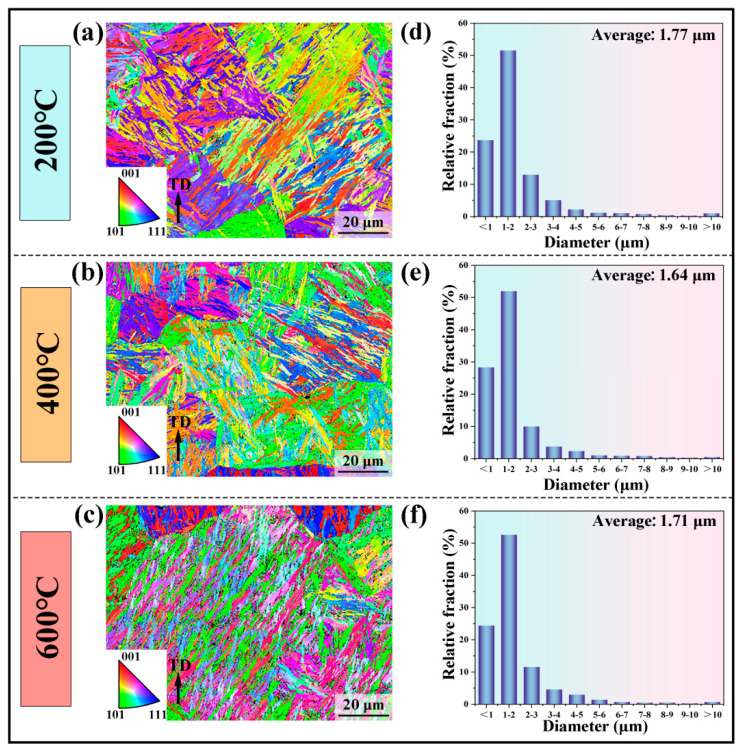
EBSD analysis of H13 steel specimens processed at different interlayer temperatures: (**a**–**c**) IPF maps; (**d**–**f**) statistical analysis of martensite size distribution.

The influence of interlayer temperature on the crystal structure was examined by EBSD analysis. The inverse pole figure (IPF) maps shown in Figure 7a–c demonstrate that the interlayer temperature affects the morphology of the martensitic microstructure. The martensitic structure is the coarsest at an interlayer temperature of 200 °C, whereas the samples fabricated at 400 °C and 600 °C exhibit a certain degree of refinement. As quantified in Figure 7d–f, the average martensite sizes corresponding to interlayer temperatures of 200 °C, 400 °C, and 600 °C are 1.77 μm, 1.64 μm, and 1.71 μm, respectively.

**Figure 8:**
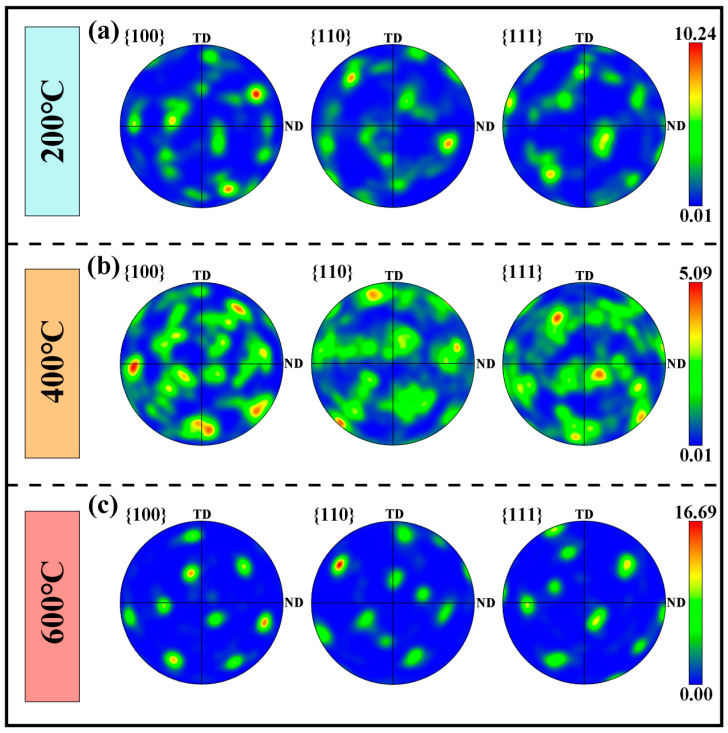
Polar coordinate plots of samples deposited by different interlayer temperature processes: (**a**) 200 °C; (**b**) 400 °C; (**c**) 600 °C.

Furthermore, the pole figures (PF) derived from EBSD data were plotted to analyze the effect of interlayer temperature on crystallographic texture, as shown in Figure 8. In the samples fabricated at interlayer temperatures of 200 °C and 400 °C, the {001} planes of the body-centered cubic (bcc) structure exhibited relatively high texture intensities. In contrast, in the sample produced at 600 °C, the bcc {110} plane exhibited the highest texture intensity, with maximum pole densities of 10.24, 5.09, and 16.69 for 200 °C, 400 °C, and 600 °C, respectively, indicating that the 600 °C sample possessed the strongest texture. Previous studies have reported that multiple variant selection during the martensitic transformation tends to randomize crystal orientations, thereby weakening the preferred orientation inherited from the parent austenite and resulting in generally weak textures in martensitic structures [31,32]. However, in this study, the α phase in the samples, fabricated at 400 °C and 600 °C, still exhibited relatively high pole densities. This behavior is attributed to the larger lath martensite size, which reduces the number of martensite variants captured within the EBSD scan area [33]. Consequently, several variants with similar orientations are overrepresented statistically, leading to higher pole densities in the PF.

**Figure 9:**
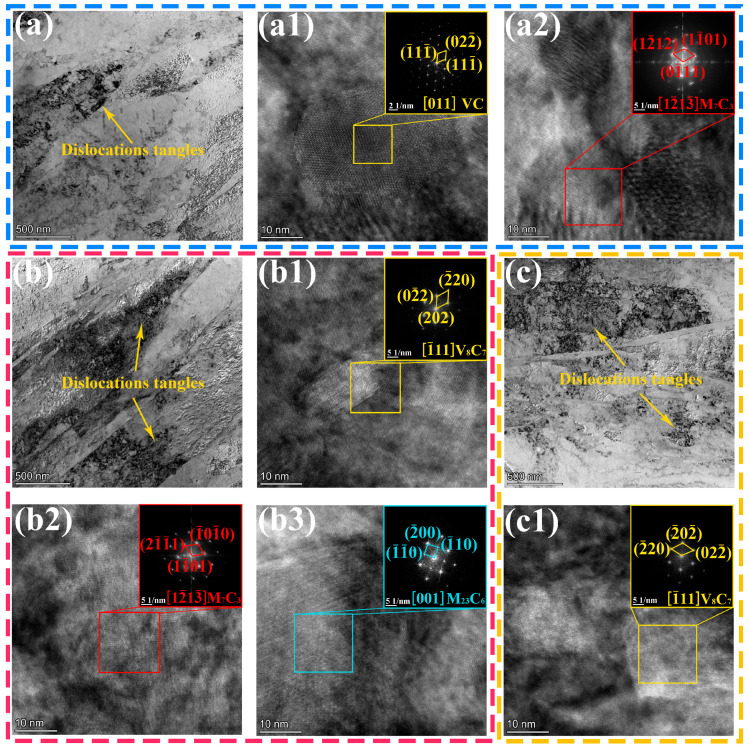
TEM results of dislocations and precipitates of H13 steel specimens at different interlayer temperatures: (**a**–**a2**) 200 °C; (**b**–**b3**) 400 °C; (**c**,**c1**) 600 °C.

In H13 tool steel, the type, quantity, size, morphology, and distribution of carbides play a critical role in determining mechanical properties such as strength, hardness, and toughness. The microstructural features and types of nanoscale carbides in H13 steel samples fabricated at different interlayer temperatures were examined using TEM, as shown in Figure 9. The bright-field images in Figure 9a–c indicate that the dislocation entanglement density in the sample processed at an interlayer temperature of 200 °C is considerably lower than that in samples processed at 400 °C and 600 °C. This observation is consistent with the XRD analysis in Figure 5 and the EBSD analysis in Figure 6, collectively highlighting the significant influence of interlayer temperature on dislocation density. Importantly, high-resolution TEM (HRTEM), combined with fast Fourier transform (FFT) analysis, revealed distinct nanoscale carbide precipitation regimes across interlayer temperatures: at 200 °C (Figure 9(a1,a2)), predominantly V-rich V_8_C_7_-type and Cr-rich M_7_C_3_ carbides; at 400 °C (Figure 9(b1–b3)), V-rich VC-type, Cr-rich M_7_C_3_, and Cr-Mo-Fe-rich M_23_C_6_ carbides; and at 600 °C (Figure 9(c1)), exclusively V-enriched VC-type carbides.

### 4.2. Effect of Interlayer Temperature on Mechanical Properties

**Figure 10:**
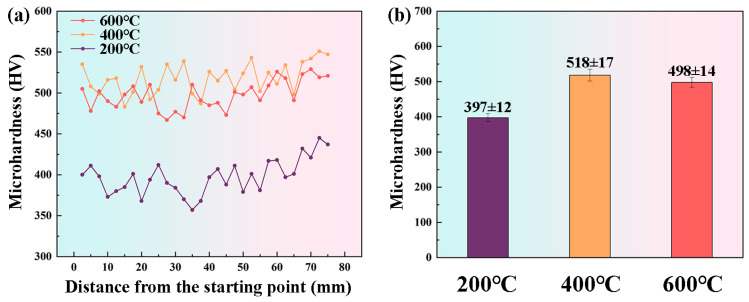
Hardness distribution profiles of specimens deposited at interlayer temperatures of 200 °C, 400 °C, and 600 °C: (**a**) hardness profile along the build direction; (**b**) average hardness bar chart.

Figure 10 presents the hardness distribution along the build direction and the statistically averaged hardness values for specimens fabricated at different interlayer temperatures (200 °C, 400 °C, and 600 °C). As shown in Figure 10a, the samples deposited at interlayer temperatures of 400 °C and 600 °C exhibit substantially higher overall hardness compared to those prepared at 200 °C. Notably, the samples deposited at 400 °C achieve the highest hardness among all the conditions evaluated. The statistical results in Figure 10b indicate that the average hardness of the samples deposited at 200 °C is 397 ± 12 HV, whereas the average hardness values for samples processed at 400 °C and 600 °C increase to 518 ± 17 HV and 498 ± 14 HV, respectively, corresponding to enhancements of 31.5% and 26.2% relative to the 200 °C specimens.

**Figure 11:**
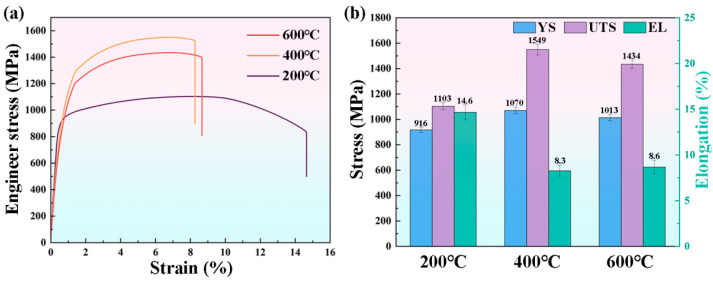
Tensile properties of specimens deposited at interlayer temperatures of 200 °C, 400 °C, and 600 °C: (**a**) stress–strain curves; (**b**) statistical histogram of YS, YTS, and EL.

Figure 11a presents the engineering stress–strain curves of as-deposited specimens, while Figure 11b comparatively illustrates the yield strength (YS), ultimate tensile strength (UTS), and elongation (EL) through bar charts. Table 3 provides a detailed summary of the tensile properties of H13 steel specimens fabricated by the WA-DED process at interlayer temperatures of 200 °C, 400 °C, and 600 °C. Specimens processed at 200 °C exhibited YS, UTS, and EL values of 916 ± 21 MPa, 1103 ± 28 MPa, and 14.6 ± 0.8%, respectively. The corresponding values for specimens processed at 400 °C were 1070 ± 25 MPa (YS), 1549 ± 43 MPa (UTS), and 8.3 ± 0.5% (EL), whereas those for specimens processed at 600 °C were 1013 ± 24 MPa (YS), 1434 ± 33 MPa (UTS), and 8.6 ± 0.7% (EL). Compared to the 200 °C samples, the YS and UTS of the 400 °C specimens increased by 16.8% and 40.4%, respectively, while EL decreased by 6.3%. Similarly, the 600 °C specimens exhibited increases of 10.5% in YS and 30.0% in UTS, accompanied by a 6% reduction in EL. The fundamental mechanisms underlying the variation in YS with interlayer temperature will be systematically discussed in subsequent sections.

**Figure 12:**
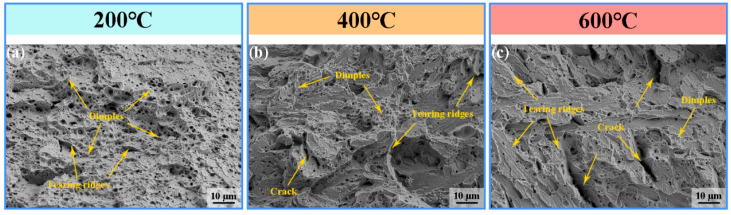
Tensile fracture surface morphology of H13 steel specimens fabricated at different interlayer temperatures: (**a**) 200 °C; (**b**) 400 °C; (**c**) 600 °C.

Figure 12 presents the SEM images of the tensile fracture surfaces of specimens fabricated at different interlayer temperatures (200 °C, 400 °C, and 600 °C). At 200 °C (Figure 12a), the specimen exhibits pronounced necking, with the fracture surface predominantly composed of numerous dimples and a few tearing ridges, indicating a primarily ductile fracture. This observation is consistent with the high EL reported in Figure 11. When the interlayer temperature is increased to 400 °C (Figure 12b), the fracture morphology evolves into a mixed mode, characterized by a reduction in dimples, an increase in tearing ridges, and the appearance of minor cracks. At 600 °C (Figure 12c), the fracture morphology undergoes a substantial transformation: dimples are largely suppressed, while tearing ridges and cracks become more prominent, indicating a transition toward a predominantly brittle fracture mode. Correspondingly, compared to specimens deposited at 400 °C, the UTS decreases.

## 5. Discussion

### 5.1. Effect of Interlayer Temperature on Strengthening Mechanisms

This study successfully produced H13 steel specimens with markedly different mechanical properties by controlling the interlayer temperature during the WA-DED process. Understanding the microstructural mechanisms underlying these variations is essential for optimizing WA-DED process parameters. It is well established that the solution strengthening, grain boundary strengthening, dislocation strengthening, and precipitation strengthening constitute the primary mechanisms governing the yield strength (YS) of metallic materials [34,35]. In general, the theoretical yield strength (*σ_y_*) can be regarded as a linear superposition of these contributions [30], with its equation as follows:

(7) $$\sigma_{y}=\sigma_{0}+\sigma_{ss}+\sigma_{gb}+\sigma_{d}+\sigma_{p},$$

where *σ*_0_ represents the intrinsic lattice strength of steel (Peierls–Nabarro stress), while *σ_ss_*, *σ_gb_*, *σ_d_*, and *σ_p_* correspond to the contributions from solid solution strengthening, grain boundary strengthening, dislocation strengthening, and precipitation strengthening, respectively. The intrinsic lattice strength *σ*_0_ can be estimated using the following equation [36]:

(8) $$\sigma_{0}=78-0.023\times T,$$

where *T* denotes the thermodynamic temperature in kelvins, set at 298 K. The calculated intrinsic lattice strength, *σ*_0_, is 71 MPa. The contributions from each strengthening mechanism are subsequently computed as explained below.

#### 5.1.1. Grain Boundary Strengthening

Grain boundary strengthening is a widely recognized mechanism in metallic materials, in which grain boundaries act as barriers to dislocation motion, thereby enhancing tensile properties. The contribution of grain boundary strengthening to the yield strength can be calculated using the Hall–Petch relationship [37]:

(9) $$\sigma_{gb}=kd^{-1/2},$$

where *k* denotes the Hall–Petch constant for H13 steel (300 MPa μm^1/2^) [38], and *d* represents the average grain size. Since the martensite block size can be considered as the effective grain dimension [39], the average block sizes measured via EBSD in Section 4.1 were directly adopted. The samples fabricated at interlayer temperatures of 200 °C, 400 °C, and 600 °C exhibited average grain sizes of 1.77 μm, 1.64 μm, and 1.71 μm, respectively. The corresponding grain boundary strengthening contributions, *σ_gb_*, were calculated as 226 MPa, 234 MPa, and 229 MPa.

Grain refinement increases the density of grain boundaries per unit volume, which act as effective barriers to dislocation motion [40]. During the tensile deformation, the grain boundaries facilitate the redistribution of stress across multiple grains, thereby reducing localized stress concentrations and delaying both crack initiation and propagation. Based on the EBSD results presented in Section 4.1, the martensite size exhibits limited sensitivity to interlayer temperature, resulting in only minor variations in the grain boundary strengthening contribution.

#### 5.1.2. Dislocation Strengthening

The contribution of dislocation strengthening to the YS can be estimated using the Bailey–Hirsch equation [41]:

(10) $$\sigma_{d}=\alpha_{d}MGb\rho^{1/2},$$

where *α_d_* is the empirical constant representing dislocation interaction strength, conventionally taken as 0.33 for body-centered cubic (BCC) lattices [42], *M* is the Taylor factor (3) [31,43], *G* signifies the shear modulus (70 GPa) [37], *b* is the magnitude of the Burgers vector (0.248 nm), and *ρ* corresponds to the dislocation density determined from XRD analysis (Figure 5). Specimens fabricated at interlayer temperatures of 200 °C, 400 °C, and 600 °C exhibit dislocation densities *ρ* of 6.23 × 10^14^ m^−2^, 9.07 × 10^14^ m^−2^, and 8.21 × 10^14^ m^−2^, respectively. Substituting these *ρ* values into equation (10), the dislocation strengthening contributions of the specimens at interlayer temperatures of 200 °C, 400 °C, and 600 °C in the YS can be obtained as 429 MPa, 518 MPa, and 492 MPa, respectively.

This strengthening originates from the mutual entanglement of dislocations during deformation, which impedes subsequent dislocation glide. As revealed by the XRD and KAM analyses presented in Section 4, the sample fabricated at an interlayer temperature of 400 °C exhibits a markedly higher dislocation density than those processed at 200 °C and 600 °C. This increase in dislocation density effectively enhances the tensile strength, indicating that dislocation strengthening is the dominant mechanism responsible for the observed improvement.

#### 5.1.3. Precipitation Strengthening

The contribution of precipitation strengthening to the yield strength can be estimated using the Orowan bypass equation [42]:

(11) $$\sigma_{p}=\frac{0.538Gbf_{v}^{1/2}}{d_{p}}\times ln\left(\frac{d_{p}}{2b}\right),$$

where *f_v_* represents the carbide volume fraction, and *d_p_* denotes the average precipitate diameter. The strengthening contribution from the Orowan mechanism decreases with increasing precipitate size. Accordingly, the coarse carbides observed in this study, owing to their large dimensions and low number density, provide an insignificant contribution to the overall strength and are thus omitted from the strengthening evaluation.

The average sizes and volume fractions of nanoscale carbides, determined via the quantitative analysis, are summarized in Table 4. The carbide volume fraction *f* was measured using the McCall–Boyd method [44]:

(12) $$f_{v}=\frac{1.4\pi nd_{p}^{2}}{6S},$$

where *n* represents the number of carbides, *d_p_* denotes the average carbide diameter, and *S* corresponds to the measurement area. The average precipitate sizes and volume fractions are summarized in Table 4. The calculated contributions of precipitation strengthening for specimens fabricated at interlayer temperatures of 200 °C, 400 °C, and 600 °C are 205 MPa, 319 MPa, and 243 MPa, respectively.

**Figure 13:**
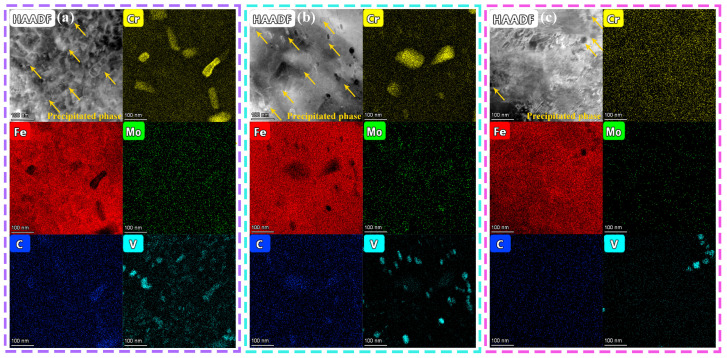
HAADF-STEM images and corresponding EDS mapping distributions of nanoscale precipitates within H13 steel specimens fabricated at different interlayer temperatures: (**a**) 200 °C; (**b**) 400 °C; (**c**) 600 °C.

In this study, the high-angle annular dark-field scanning transmission electron microscopy (HAADF-STEM) coupled with energy-dispersive spectroscopy (EDS) was employed to characterize the primary features of the precipitates. Figure 13 presents the HAADF-STEM images and corresponding EDS analyses of the samples fabricated at three different interlayer temperatures. The EDS results confirm that the precipitated phases formed at interlayer temperatures of 200 °C and 400 °C are predominantly composed of Cr, V, Mo, and C. In contrast, the precipitates formed at an interlayer temperature of 600 °C consist mainly of V and C. These findings corroborate the phase identification results presented in Figure 9. Notably, within the investigated processing window, the sample fabricated at an interlayer temperature of 400 °C exhibits the most uniform and favorable precipitate dispersion.

**Figure 14:**
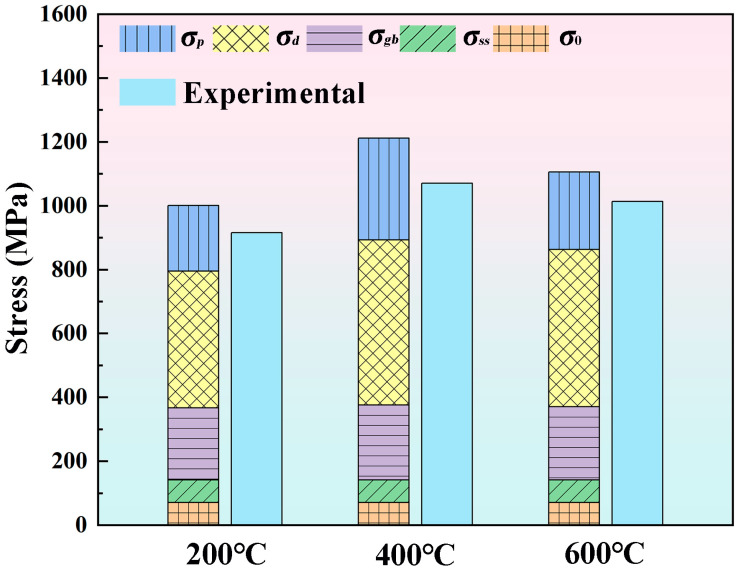
Comparison of quantitatively assessed contributions from various enhancements with experimental findings.

Figure 14 quantitatively presents the contributions of different strengthening mechanisms to the YS of the three specimens, alongside the corresponding experimental data. Notably, the solid solution strengthening contribution in martensitic steel is taken as *σ_ss_* = 70 MPa [45]. The quantitative analysis indicates that dislocation strengthening exerts the most significant effect, followed by precipitation strengthening. Grain boundary strengthening provides a moderate yet meaningful contribution, whereas the solid solution strengthening remains relatively constant across the specimens. A comparison between the theoretical calculations and experimental results demonstrates a high level of agreement, confirming the reliability of the YS predictions.

### 5.2. Effect of Interlayer Temperature on Microstructural Evolution in WA-DED

The WA-DED process is inherently layer-by-layer, giving rise to complex thermal cycling. Rapid melting and solidification induced by the arc heat input result in substantial heat accumulation and repeated reheating of previously deposited layers. Consequently, the interlayer temperature governs the thermal history of the deposited material, thereby controlling its subsequent microstructural evolution.

**Figure 15:**
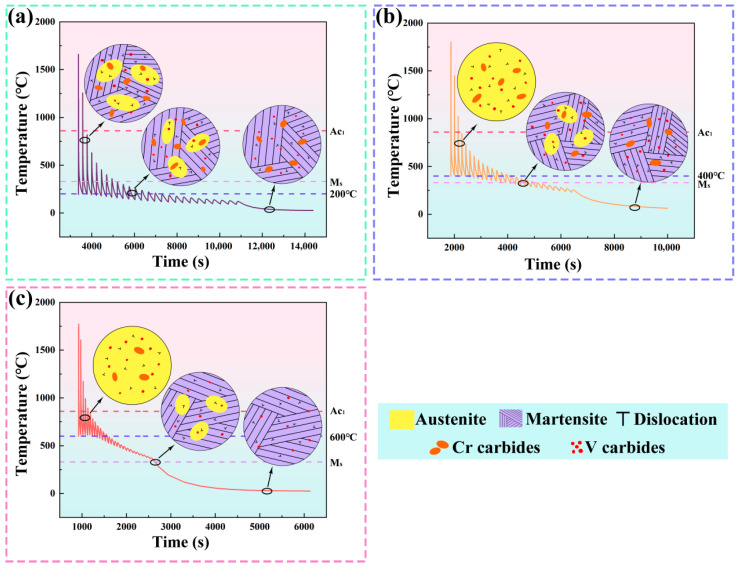
Simulated thermal cycling and corresponding microstructure evolution of H13 steel during WA-DED at different interlayer temperatures: (**a**) 200 °C; (**b**) 400 °C; (**c**) 600 °C.

Figure 15 illustrates the thermal cycling curves of the intermediate layer in the deposited component under different interlayer temperatures, along with the corresponding microstructural evolution. As shown in Figure 15a, an interlayer temperature of 200 °C substantially prolongs the overall deposition time, resulting in prolonged exposure of the deposited material to a thermal environment near 200 °C. This extended thermal history markedly affects the resultant microstructure. Under these conditions, the diffusion rates of alloying elements (Cr, Mo, V) within the matrix and along grain boundaries are significantly reduced, hindering the formation of fine and uniformly distributed carbides [46]. Concurrently, the limited precipitation of secondary phases diminishes their dislocation-pinning effect, thereby suppressing dislocation interactions and constraining the development of highly stressed microstructural features [47].

As illustrated in Figure 15b, when the interlayer temperature increases to 400 °C, the heat input from the subsequent deposition layer provides sufficient thermodynamic driving force for the precipitation process, facilitating the uniform formation of nanoscale carbides [48]. These finely and uniformly distributed carbide particles act as effective second-phase reinforcements, strongly pinning dislocations, impeding their glide and recovery, and consequently increasing the dislocation density [47]. Furthermore, these carbides inhibit the growth of martensite laths during subsequent cooling, leading to the development of a refined martensitic microstructure [49].

As shown in Figure 15c, when the interlayer temperature further increases to 600 °C, the deposited material remains within a high-temperature regime. Although the residence time at elevated temperature is relatively short, limiting its impact on dislocation annihilation, it still promotes recovery and recrystallization processes, leading to the rearrangement and partial annihilation of dislocations [50]. Moreover, the high-temperature environment significantly enhances the diffusivity of solute atoms. The accelerated diffusion of alloying elements facilitates the dissolution of precipitates, resulting in a pronounced reduction in the number density of the second-phase particles [48].

The interlayer temperature critically governs microstructural evolution and the associated strengthening mechanisms in WA-DED fabricated H13 steel, thereby dictating its overall mechanical performance. Specimens processed at an interlayer temperature of 200 °C exhibit coarse grains, low dislocation density, and larger precipitates with reduced volume fraction. Despite this, they retain moderate strength and relatively high EL, primarily relying on limited contributions from dislocation, precipitation, and grain boundary strengthening, which constrains the overall strength. Increasing the interlayer temperature to 400 °C enhances dislocation density, refines the grain structure, and promotes the formation of finely dispersed precipitates with elevated volume fraction. These precipitates effectively pin dislocations, enabling synergistic strengthening through combined mechanisms, thereby achieving superior strength and hardness. Further elevating the interlayer temperature to 600 °C prolongs exposure to high temperature, accelerating dislocation recovery and grain coarsening. This substantially reduces strengthening contributions, while concurrent precipitate coarsening further diminishes overall efficacy. Consequently, the interlayer temperature emerges as a critical processing parameter, exerting its influence through differential regulation of synergistic interactions under distinct thermal regimes.

## 6. Conclusions

This study systematically investigates the effect of interlayer temperature on the microstructure and mechanical properties of H13 steel fabricated via WA-DED. The corresponding thermal cycling processes were simulated using Abaqus software. The research elucidates the intrinsic relationship between the microstructural evolution and the mechanical behavior of specimens subjected to different thermal histories. The findings demonstrate that interlayer temperature plays a pivotal role in governing both the microstructure and mechanical performance of WA-DED components. The main conclusions are summarized as follows:

1. The samples fabricated at interlayer temperatures of 200 °C, 400 °C, and 600 °C are composed of martensite and carbides. The observed variations in yield strength are primarily ascribed to the combined effects of martensite size, carbide distribution and morphology, and dislocation density. Among these strengthening mechanisms, dislocation strengthening exerts the most pronounced influence on the yield strength.
2. Microstructural analysis reveals that at an interlayer temperature of 200 °C, both martensite and carbide structures are relatively coarse, and the dislocation density is the lowest. Increasing the interlayer temperature to 400 °C results in the finest martensitic structure, the highest dislocation density, and uniformly dispersed fine carbides. Further elevation of the interlayer temperature to 600 °C leads to a reduction in carbide volume fraction and a decrease in dislocation density compared to the 400 °C condition.
3. A comprehensive analysis of the mechanical properties indicates that the sample fabricated at an interlayer temperature of 400 °C exhibits optimal strength, with a UTS of 1549 MPa and a YS of 1070 MPa. These values exceed those of the samples prepared at 200 °C (UTS = 1103 MPa, YS = 916 MPa) and 600 °C (UTS = 1434 MPa, YS = 1013 MPa). However, the elongation of the 400 °C sample (EL = 8.3%) is slightly lower than that of the specimens deposited at 200 °C (EL = 14.6%) and 600 °C (EL = 8.6%).

## Figures and Tables

**Table 1:** Chemical composition of H13 steel substrate and wire feedstock (wt.%).

H13	C	Cr	Mo	V	Mn	Si	Fe
Base metal	0.39	5.00	1.22	0.86	0.38	0.83	Bal.
Wire	0.39	4.87	1.34	0.84	0.33	0.93	Bal.

**Table 2:** WA-DED processing parameters for H13 specimens.

Parameter	Value
Current (A)	136
Voltage (V)	19.2
Wire Feeding Speed (m/min)	8
Welding Speed (mm/s)	3.5
Increment of *Z*-axis (mm)	1.5
Shielding gas type	90% Ar + 10% CO_2_
Shielding gas flow (L/min)	20
Substrate temperature (°C)	25
Interlayer temperature (°C)	200, 400, 600

**Table 3:** Mechanical properties of H13 steel specimens at different interlayer temperatures.

Samples	YS (MPa)	UTS (MPa)	EL (%)
200 °C	916 ± 21	1103 ± 28	14.6 ± 0.8
400 °C	1070 ± 25	1549 ± 43	8.3 ± 0.5
600 °C	1013 ± 24	1434 ± 33	8.6 ± 0.7

**Table 4:** Quantitative distribution of nanoscale carbides in as-deposited H13 tool steel specimens.

Zones of Samples	200 °C	400 °C	600 °C
Average diameters (nm)	34.5	20.8	21.5
Volume fraction (%)	3.2	3.6	2.2

## Data Availability

The original contributions presented in this study are included in the article. Further inquiries can be directed to the corresponding authors.
