# Correction: Li et al. Effect of Interlayer Temperature-Controlled Thermal Cycling on the Microstructure and Mechanical Properties of Wire Arc Directed Energy Deposition H13 Steel. *Materials* 2026, *19*, 111

**DOI:** 10.3390/ma19112300

**Published:** 2026-05-29

**Authors:** Chuang Li, Hawke Suen, Yajin Yang, Liang Zhang, Qiuxia Chen, Tianlong Gao, Bo Yuan, Lyusha Cheng, Zhe Lv

**Affiliations:** 1 School of Materials and Metallurgy, University of Science and Technology Liaoning, Anshan 114051, China; lc042907@163.com; 2 Institute of Intelligent Manufacturing Technology, Shenzhen Polytechnic University, Shenzhen 518055, China; zhangliang@szpu.edu.cn (L.Z.); gtlzs18@163.com (T.G.); yuanbo@szpu.edu.cn (B.Y.); 3 Shenzhen Institute of Advanced Technology, Chinese Academy of Sciences, Shenzhen 518055, China; 4 School of Science, Harbin Institute of Technology, Shenzhen 518055, China; yangyajin1120@126.com; 5 School of Artificial Intelligence, Shenzhen Polytechnic University, Shenzhen 518055, China; chenqiuxia@szpu.edu.cn; 6 Department of Mechanical and Electrical Engineering, Shenzhen Polytechnic University, Shenzhen 518055, China

In the original publication [1], there were overlaps in Figure 6 as published. The corrected Figure 6 appears below. The authors state that the scientific conclusions are unaffected. This correction was approved by the Academic Editor. The original publication has also been updated.

## Figures and Tables

**Figure 6:**
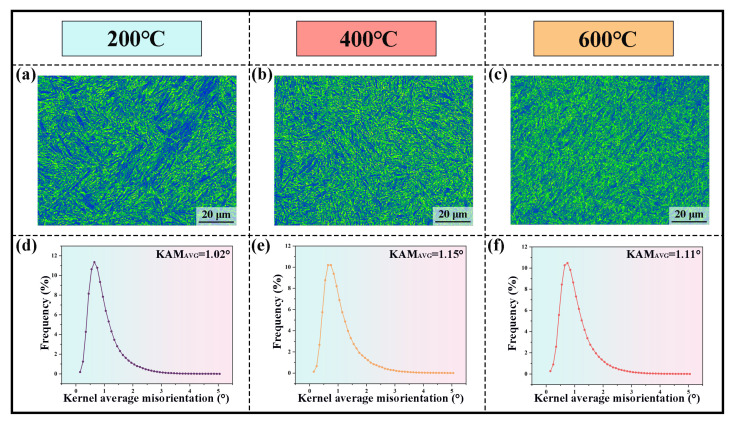
KAM images of H13 steel samples processed at different interlayer temperatures: (**a**,**d**) 200 °C; (**b**,**e**) 400 °C; (**c**,**f**) 600 °C.
